# Real-world Health Data and Precision for the Diagnosis of Acute Kidney Injury, Acute-on-Chronic Kidney Disease, and Chronic Kidney Disease: Observational Study

**DOI:** 10.2196/31356

**Published:** 2022-01-25

**Authors:** Karen Triep, Alexander Benedikt Leichtle, Martin Meister, Georg Martin Fiedler, Olga Endrich

**Affiliations:** 1 Medical Directorate, Medizincontrolling Inselspital, University Hospital Bern Insel Gruppe Bern Switzerland; 2 Insel Data Science Center Inselspital, University Hospital Bern Insel Gruppe Bern Switzerland; 3 Directorate of Technology and Innovation Inselspital, University Hospital Bern Insel Gruppe Bern Switzerland; 4 University Institute of Clinical Chemistry Inselspital, University Hospital Bern Insel Gruppe Bern Switzerland

**Keywords:** acute kidney injury, chronic kidney disease, acute-on-chronic, real-world health data, clinical decision support, KDIGO, ICD coding

## Abstract

**Background:**

The criteria for the diagnosis of kidney disease outlined in the Kidney Disease: Improving Global Outcomes guidelines are based on a patient’s current, historical, and baseline data. The diagnosis of acute kidney injury, chronic kidney disease, and acute-on-chronic kidney disease requires previous measurements of creatinine, back-calculation, and the interpretation of several laboratory values over a certain period. Diagnoses may be hindered by unclear definitions of the individual creatinine baseline and rough ranges of normal values that are set without adjusting for age, ethnicity, comorbidities, and treatment. The classification of correct diagnoses and sufficient staging improves coding, data quality, reimbursement, the choice of therapeutic approach, and a patient’s outcome.

**Objective:**

In this study, we aim to apply a data-driven approach to assign diagnoses of acute, chronic, and acute-on-chronic kidney diseases with the help of a complex rule engine.

**Methods:**

Real-time and retrospective data from the hospital’s clinical data warehouse of inpatient and outpatient cases treated between 2014 and 2019 were used. Delta serum creatinine, baseline values, and admission and discharge data were analyzed. A Kidney Disease: Improving Global Outcomes–based SQL algorithm applied specific diagnosis-based International Classification of Diseases (ICD) codes to inpatient stays. Text mining on discharge documentation was also conducted to measure the effects on diagnosis.

**Results:**

We show that this approach yielded an increased number of diagnoses (4491 cases in 2014 vs 11,124 cases of ICD-coded kidney disease and injury in 2019) and higher precision in documentation and coding. The percentage of unspecific ICD N19-coded diagnoses of N19 codes generated dropped from 19.71% (1544/7833) in 2016 to 4.38% (416/9501) in 2019. The percentage of specific ICD N18-coded diagnoses of N19 codes generated increased from 50.1% (3924/7833) in 2016 to 62.04% (5894/9501) in 2019.

**Conclusions:**

Our data-driven method supports the process and reliability of diagnosis and staging and improves the quality of documentation and data. Measuring patient outcomes will be the next step in this project.

## Introduction

### Background

Many definitions of diagnoses are rule-based and contain complex algorithms. This applies in particular to the diagnoses of kidney injury and kidney disease (KD). For example, the diagnosis of acute kidney injury (AKI) stage 3 according to the Kidney Disease: Improving Global Outcomes (KDIGO) guidelines is defined as follows: an increase in serum creatinine (SCr) from under 4 mg/dL (353.6 µmol/L) to over 4 mg/dL within 7 days or an increase of SCr by 200% or more within 7 days. The increase and decrease have to be considered as follows: the gradient of increase versus the absolute increase, the increase versus decrease, and the highest stage. Moreover, the SCr baseline calculation has to be conducted: the lowest value during hospitalization or the arithmetic mean of all outpatient measurements before the index admission.

With the increasing availability of health data, automatic deducing of complex diagnoses has become possible. Correctly assigning diagnoses requires high precision, validity and reliability, the varying interrater reliability of diagnosis and the International Classification of Diseases (ICD) coding, affecting accuracy [1-4]. Interrater reliability shows insufficient values for certain diagnoses when comparing ICD codes or patients’ records of the diagnoses of AKI and chronic KD (CKD) [5,6].

The global burden of KDs is high. Using a modification of the original glomerular filtration rate (GFR) estimating equation (the Chronic Kidney Disease Epidemiology Collaboration [CKD-EPI] equation), it was discovered that 11.6% of the adult residents in the United States have CKD stages 1-4, and its prevalence has increased over the past decade. Similar figures have been reported in several other countries [7-10]. In 2009, the US Renal Data System estimated that depending on the estimating equations used, the prevalence of CKD had increased by 20%-25% over the preceding decade [11].

The diagnoses of AKI, CKD, and acute-on-chronic KD are highly relevant as a comorbidity, intercurrent disease, or complication [12,13]. Inpatients with KD and kidney injury show a higher mortality and the staging implies an impact on outcomes [14,15]. The 2012 KDIGO Clinical Practice Guideline for AKI [16] and the Clinical Practice Guideline for the Evaluation and Management of CKD [17] offer guidelines containing definitions and classifications; ongoing areas of controversies and limitations of the evidence are also discussed in these documents. The definitions of AKI and CKD require a complex analysis of a patient’s recent and historical laboratory values, a time-consuming process impeded by missing values and prone to errors if conducted manually. Misclassification impairs the choice of therapeutic approach, outcomes, high-quality documentation, data validity, and reimbursement. Moreover, an unclear definition of the individual creatinine baseline level and the approximate ranges of normal values without adjusting for age, ethnicity, comorbidities, and treatment aggravate the difficulties of diagnosis [18-27].

Clinical decision support systems can provide a systematic and objective way to enhance complex reasoning related to differential diagnostics. They can facilitate the process of diagnosis, contributing to its reliability [28-33]. Accumulating health data enables the providers to access relevant information for timely diagnosis, supporting effective management throughout care [10,34]. In recent times, national health systems, such as the National Health Service (NHS), have started supporting more advanced approaches for detecting patients with kidney injuries [35].

In Switzerland, since 2017, based on the official coding rules, AKI and CKD have been coded according to the KDIGO classification. However, documentation of the exact staging is often missing in the discharge documentation in many cases.

At our hospital (quaternary care university level), KD shows a rising relevance because the prevalence of patients with a GFR of <60 ml/min measured has been increasing during the recent years. Moreover, the ICD diagnoses of KD are relevant for reimbursement. Nevertheless, many inpatient cases with a GFR of <60 ml/min were not ICD-coded for any KD, and a clinical decision support has not yet been implemented.

### Objectives

This study aims to evaluate a novel data-driven method to assign highly specific diagnoses of AKI and CKD by extracting historical and real-time data from the hospital’s data warehouse. We hypothesize that by using a data-driven approach of diagnosis on routinely collected laboratory values, we can improve the detection and precision of the diagnosis and staging of AKI and CKD.

## Methods

### Study Population and Setting

Administrative and laboratory data of all inpatient and outpatient cases were used (Inselspital University Hospital Bern, 2014-2019; all Insel Gruppe, Bern, 2016-2019 with 200,000 inpatient and outpatient cases per year, of which, approximately 62,000 inpatient cases had ICD-coded diagnoses). Data from 2014 to 2016 were used for benchmarking purposes as a baseline at the start of the study (2017). Test data sets of cases from 2016 were used to evaluate the accuracy of the algorithm. The data for measuring the impact were selected from 2017 to 2019.

### Definition of AKI

According to the KDIGO Clinical Practice Guidelines (2012) for AKI [16], we defined and staged AKI as follows (plasma creatinine instead of SCr):

- Stage 3: increase of SCr from under 4 mg/dL (353.6 µmol/L) to over 4 mg/dL within 7 days
- Stage 3: increase of SCr by 200% or more within 7 days
- Stage 2: increase of SCr by 100%-200% within 7 days
- Stage 1: increase of SCr by 50%-100% within 7 days
- Stage 1: increase of SCr by 0.3 mg/dL (26.52 µmol/L) within 48 hours

The decrease in SCr to baseline levels after starting the in-hospital measurement was interpreted as suggested by the KDIGO guidelines. With several positive findings, the gradient of the increase versus the absolute increase, the increase versus decrease, and the highest stage were prioritized for applying the specific stage. All available SCr measurements, along with date and time stamps were used. Inpatients with no available SCr measurements were classified as not having AKI.

### Oliguria

Oliguria is still a controversial diagnostic criterion with regard to definition and practice of measurement, especially outside the intensive care setting. The hourly urine output data required to determine oliguria within any 6-, 12-, or 24-hour window is not reliably captured in the non–intensive care unit setting [25]. Therefore, we did not include it in the AKI definition of this study, which was consistent with the NHS England National Patient Safety Alert [35,36].

### Baseline Definition

The baseline estimation was not specifically defined by the KDIGO guidelines; however, several methods were compared for baseline estimation [16,19,21,24,25,37].

We defined the baseline value for AKI as either the lowest value during hospitalization or the arithmetic mean of all outpatient SCr measurements 90 days before the index admission, if available, and took the lowest values for diagnosis. Either one may reasonably reflect the patient’s premorbid baseline. Using the values at admission was considered; however, although the values may be the lowest for community-acquired AKI, they may be missing. Different approaches were not compared in this study.

### Definition of CKD

In this study, a possible CKD was defined according to the KDIGO Clinical Practice Guideline for the Evaluation and Management (2012) of CKD [17], that is, a decreased GFR of <60 mL/min/1.73 m^2^, an albumin creatinine ratio (ACR) of >30 mg/g (>3 mg/mmol), or a history of kidney transplantation (estimated according to the CKD-EPI equation). GFR categories were assigned as follows:

- Stage 5: all values under 15 mL/min/1.73 m^2^ for >91 days
- Stage 4: all values under 30 mL/min/1.73 m^2^ for >91 days
- Stage 3: all values under 60 mL/min/1.73 m^2^ for >91 days
- Stage 2: all values under 90 mL/min/1.73 m^2^ for >91 days

### Definition of ACR

In addition, according to the KDIGO criteria we integrated albuminuria into the model as a marker of kidney damage, related to mortality and kidney outcome in CKD [17,37], using the ACR values, as follows:

- Severely increased: SCr (µmol/L)/albumin (g/L) >30 mg/g
- Moderately increased: SCr (µmol/L)/albumin (g/L) between 3 and 30 mg/g
- Normal to mildly increased: SCr (µmol/L)/albumin (g/L) <30 mg/g

Values from one sample or values measured within an interval of 30 days were considered.

### Architecture and Algorithm

**Figure 1:**
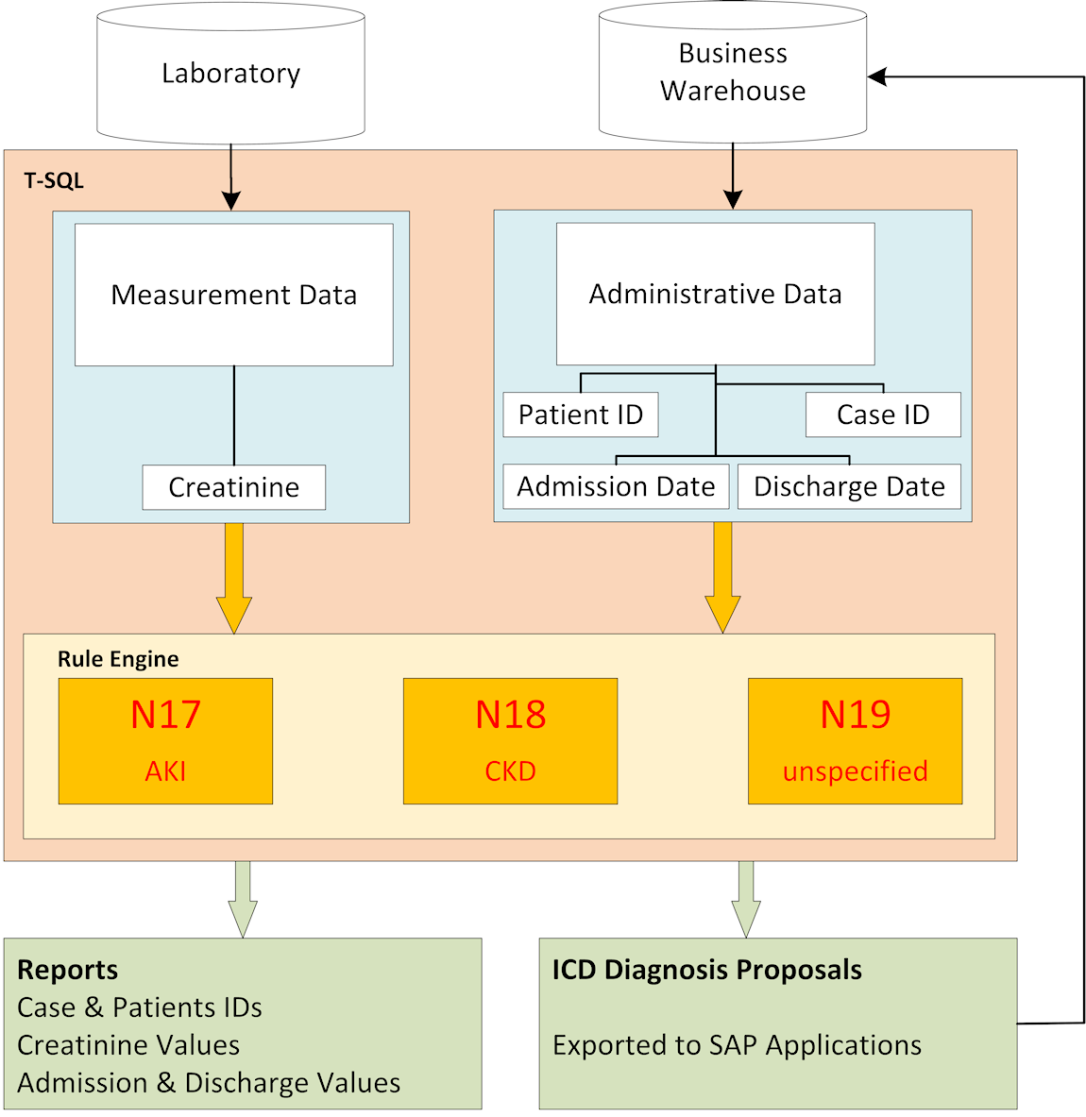
Architecture and dataflow. AKI: acute kidney injury; CKD: chronic kidney disease; ICD: International Classification of Diseases.

**Figure 2:**
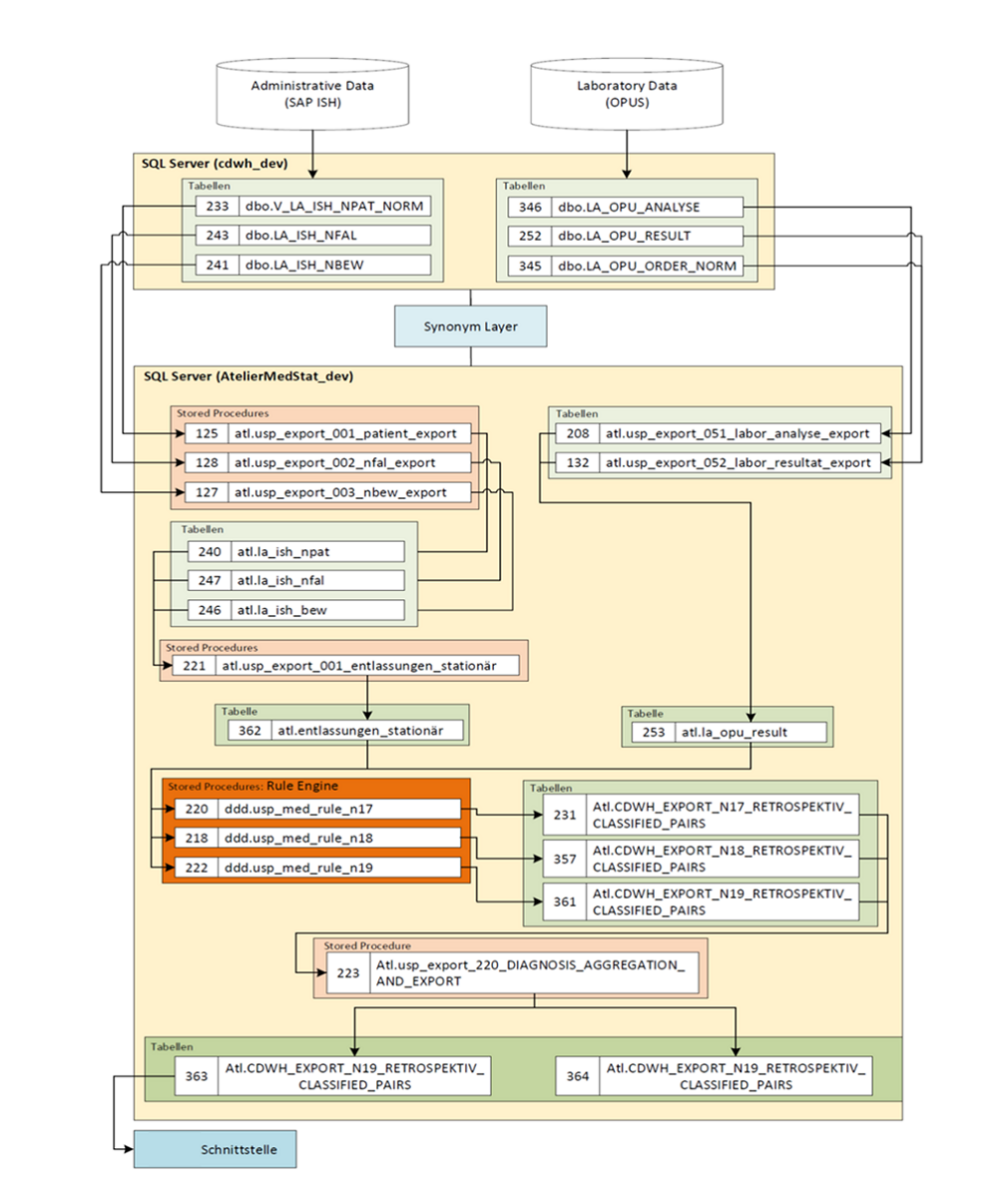
Architecture for retrospective analysis. OPUS: laboratory information system by OSM Group; SAP ISH: Systems Applications and Products in Data Processing Industry Solution Healthcare.

A complex dataflow was established to make all the required variables available for calculation. First, an SQL-based algorithm processed the data warehouse’s data (rule engine and HL7 [Health Level Seven International] messages) and detected the potential cases of KD. All available SCr measurements with date and time stamps were used. The patient identification number was defined as the primary key but was only used as a linkage code for administrative and clinical or laboratory data. Patient- and case-related laboratory and administrative historical and real-time data had to be extracted from the source systems, merged, and computed for diagnosis and stage. Second, the output of the correlating ICD [38,39] code was connected by the detection date to the distinct date of the patient’s inpatient case (the case ID linked to the entry and discharge dates related to the patient ID). Third, the test results were processed to the recipient and included a staging of AKI and CKD according to the abovementioned criteria. The architecture and dataflow of AKI and CKD are illustrated in Figure 1, and the architecture and dataflow of the retrospective calculation are shown in Figure 2.

The steps of computation were as follows: (1) for AKI, selecting inpatients hospitalized during a specified period, selecting laboratory values (SCr) 7 days before admission until discharge, mapping values from 48 hours to 7 days apart, and classifying values according to the ICD standard [38,39]; and (2) for CKD, selecting inpatients’ or outpatients’ laboratory values (estimated GFR [eGFR]), mapping the values of eGFR at least three months apart, calculating the mean minimum and maximum values of each period and the difference of the mapped values in hours, and classifying values according to the ICD standard.

**Figure 3:**
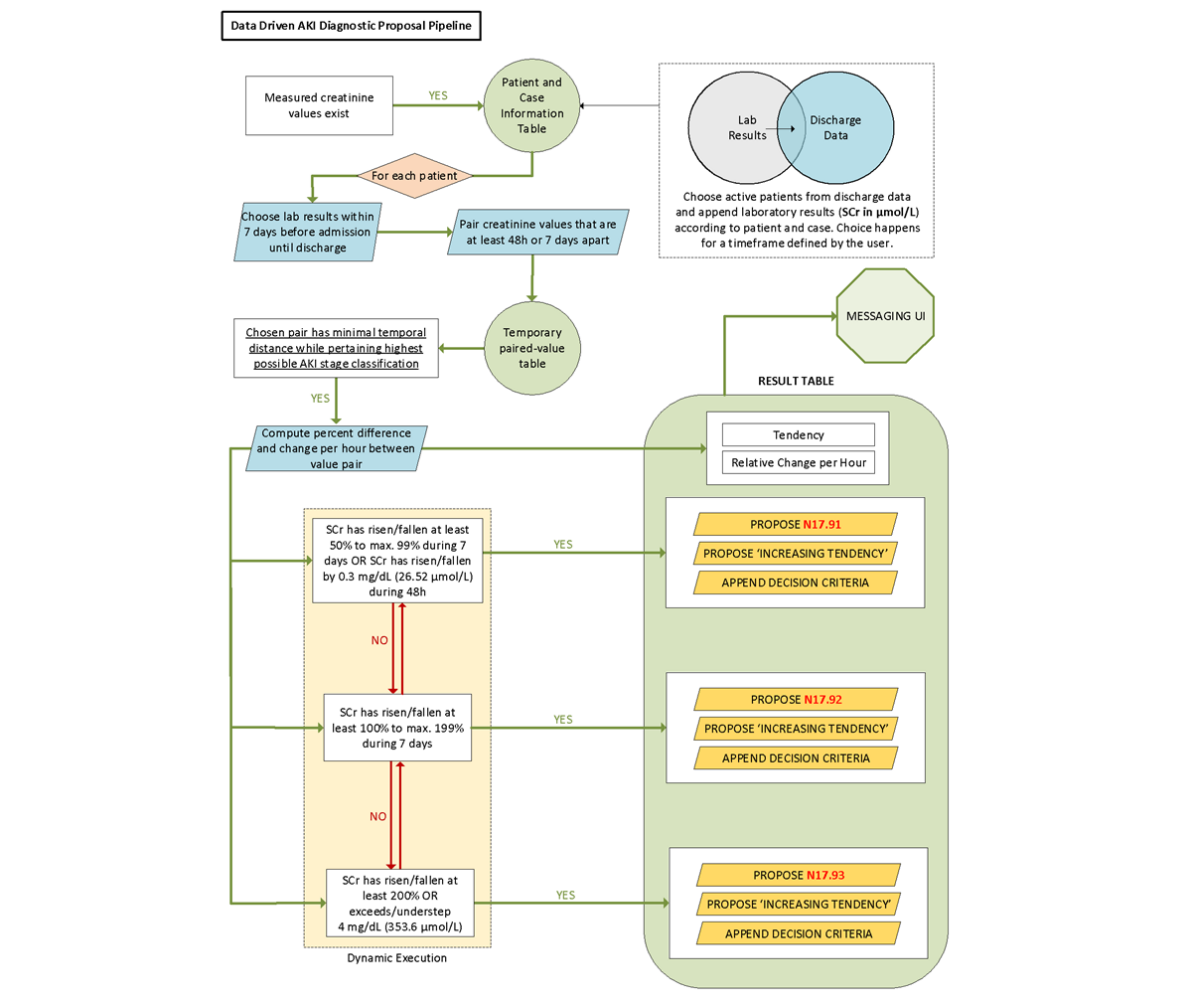
Algorithm of diagnosis of AKI. AKI: acute kidney injury; SCr: serum creatinine; UI: user interface.

The output for AKI was defined as the highest stage of diagnosis with the shortest period between the mapped values. The relevant result for CKD was defined as the highest stage of diagnosis with the longest period between mapped values. All patients with fulfilled criteria during the previous year for the specific diagnosis and with values positively corresponding to diagnosis during the last 3 months were detected. The algorithm for AKI is presented in Figure 3 and for CKD, in Multimedia Appendix 1. The algorithm was tested on testing data sets by technicians and clinicians. The algorithm was technically adjusted until all tested cases showed correct diagnoses and stages according to the formal definitions provided.

### Text Mining

A text mining pipeline was implemented using Apache Solr (The Apache Software Foundation) to compare the results of the algorithms with those of the reports. In this process, all relevant reports were loaded into a Solr collection and searched for terms, such as *AKI*, *CKD*, *eGFR*, *KDIGO*, *creatinine*, and *renal failure* (German translation: *kreatinin*, *niereninsuffizienz*, and *nierenversagen*), and the exact KDIGO staging (eg, G1A1 [GFR ≥90 ml/min per 1.73 m^2^ ACR <30 mg/g (<3mg/mmol)]).

For terms with a tilde character (“~”), a fuzzy search algorithm was applied to ensure that not only one spelling of a term was found. The Damerau-Levenshtein distance algorithm was used for this purpose. A separate CSV file was generated for each search term, including the case ID, date of report generation, and report type. Each row corresponded to a finding of the respective search term.

The CSV files were then loaded into the database containing the algorithm results and other case data. Using a transact-SQL script, the results from all sources were then aggregated at the case level. On this basis, the cases could be filtered and evaluated for constellations of interest.

### Process of Diagnosis

Being aware of the purely arithmetic method of diagnosis implemented in 2017, clinical judgment was integrated into the process, especially to verify the chronic diagnoses and distinguish between AKI and unstable CKD [40,41]. Therefore, the real-time information and retrospectively detected diagnoses were compared with the documentation in the patients’ health records using ICD diagnoses coded manually (comparison of automatically generated and manually coded ICD codes) and text mining (diagnoses and stages). Differences were then analyzed by the clinicians. Mostly, the cases with singular ICD codes relevant for reimbursement were analyzed and validated. After rejecting a certain fraction of the automatically generated diagnoses because of the clinician’s judgment or lack of documentation, the corresponding codes were deleted. Only validated diagnoses were retained in the database. The effect of the validation was monitored by analyzing the mutation of diagnoses from generated to coded ICD codes (log file of all ICD code mutations).

**Figure 4:**
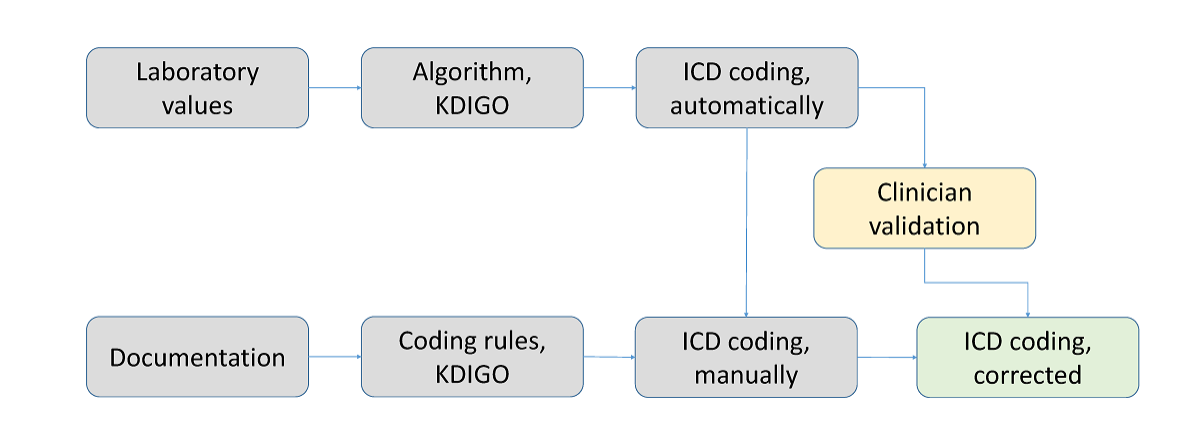
Process of diagnosis. ICD: International Classification of Diseases; KDIGO: Kidney Disease: Improving Global Outcomes.

During the period of the project, the process of diagnosis was supported by documentation templates, instruction, and close communication with the clinicians. The data processed were not used to set up an alerting system but were validated retrospectively after the patients’ discharge. The diagnosis process is illustrated in Figure 4.

### Catalogs

Bound by the Swiss regulations, the following catalogs were applied [38,39]: for the discharge year 2014, the International Statistical Classification of Diseases and Related Health Problems, 10th revision, German Modification (ICD-10-GM) 2012; from 2015 to 2016, the ICD-10-GM 2014; from 2017 to 2018, the ICD-10-GM 2016; and for 2019, the ICD-10-GM 2018. Because of a mutation in codes for AKI (ICD N17-) from 2014 to 2016, some analyses could only be conducted for the data from 2017 onward (Multimedia Appendix 2); ICD-10-GM codes catalogs from 2012-2018, effective in Switzerland from 2014 to 2020.

### Reimbursement

To measure the effect after the successful implementation of the algorithm we planned a simulation of Swiss Diagnosis Related Groups, Inpatient Tariff (SwissDRG) income of 6 months’ coding (inpatient cases from February 1, 2020, to July 31, 2020) in 2020 with and without grouping the automatically calculated ICD diagnoses; the SwissDRG web-based batch grouper version 9.0 2020/2020 was used.

### Analysis and Software

The automatically generated, previously coded, rejected, and validated ICD diagnoses were compared per code category and per specific code. The prevalence of the codes was calculated for all inpatient cases and for inpatient cases with coded KD (all diagnoses). The proportion of specific medical information (text, laboratory values, reference to KDIGO classification, and formal KDIGO staging) documented in the corresponding discharge letters was calculated.

The following software were used during analyses: Medical coding software Systems Applications and Products in Data Processing Industry Solution Healthcare (SAP IS-H), Medical Coding Tool ID Diacos, Clinical Data Phoenix CGM, Business Data Ware House SAP BW, Microsoft Excel 2010, R developing software (R version 3.5.0 2018-4-23), RStudio version 1.1.453, and RStudio Team (2016) as well as RStudio: Integrated Development for R (RStudio, Inc) and ggplot2 version 3.1.0.

### Ethics

The ethics committee of the Canton Bern approved this study (BASEC-Req-2018-01184).

## Results

### General Remarks

The method applied in this study to assign the specific diagnoses and exact stages of AKI and CKD produced highly reliable results. Moreover, the process of communicating and verifying the diagnoses improved the validity in the medical context of the individual patient. Diagnoses and stages could be displayed in near to real time and retrospective calculations could be conducted for the previous 6 years. As the algorithm considered acute and chronic diseases, this project is one of the few to integrate the diagnosis of acute-on-chronic KD. The specific diagnoses documentation and the exact staging in the patients’ discharge letters could be improved.

### Overview

An increasing prevalence of inpatient cases with a measured eGFR of <60 ml/min can be shown for the discharge years 2014-2019 (from 4362/42,703, 10.21% cases in 2014 to 12,519/66,958, 18.69% cases in 2019). The proportion of ICD-coded inpatient cases with ICD codes for any KD diagnosis in the ICD categories N17-/N18-/N19- during the same period increased for all inpatients (from 4491/42,703, 10.52% cases in 2014 to 11,124/66,958, 16.62% cases in 2019) and for the group of cases with an eGFR of <60 ml/min (from 2167/4362, 49.48% cases in 2014 to 7596/12,519, 60.68% in 2019). The proportion of coded cases of KD with an eGFR of <60 ml/min was 49.68% (2167/4362) in 2014 and 45.09% (5005/11,100) in 2016 and dropped to 60.68% (7596/12,519) in 2019. Between 2014 and 2019, the prevalence of all KD-coded cases increased from 10.52% (4491/42,703) cases in 2014 to 16.61% 11,124/66,958) cases in 2019. The main increase in the prevalence of coded cases of KD was observed between 2017 and 2019, after project initiation as shown in Table 1.

**Table 1:** Prevalence of cases with estimated glomerular filtration rates (eGFRs) of <60 ml/min and kidney injury (KI) coding (all International Classification of Diseases [ICD] codes N17-/N18-/N19-).

Distribution	Year of discharge						
	2014	2015	2016	2017	2018	2019	Total
Inpatient cases, N	42,703	45,138	64,478	65,146	66,038	66,958	350,461
Inpatient cases with measured eGFR, n (% of inpatient cases total)	20,610 (48.26)	37,326 (82.69)	40,917 (63.46)	40,109 (61.57)	41,552 (6292)	46,800 (69.89)	227,314 (64.86)
Inpatient cases with an eGFR of <60 ml/min, n (% of inpatient cases total)	4362 (10.21)	8786 (19.47)	11,100 (17.22)	10,695 (16.42)	10,570 (16.01)	12,519 (18.67)	58,032 (16.65)
Any KI-coded (ICD N17-/N18-/N19-) cases, n (%)	4491 (10.51)	4786 (10.6)	8422 (13.06)	8512 (13.06)	10,165 (15.39)	11,124 (16.61)	47,500 (13.55)
Any KI-coded (ICD N17-/N18-/N19-) inpatient cases with an eGFR of <60 ml/min, n (%)	2167 (49.68)	4029 (45.86)	5005 (45.09)	5031 (47.04)	5983 (56.6)	7596 (60.68)	29,811 (51.37)

**Figure 5:**
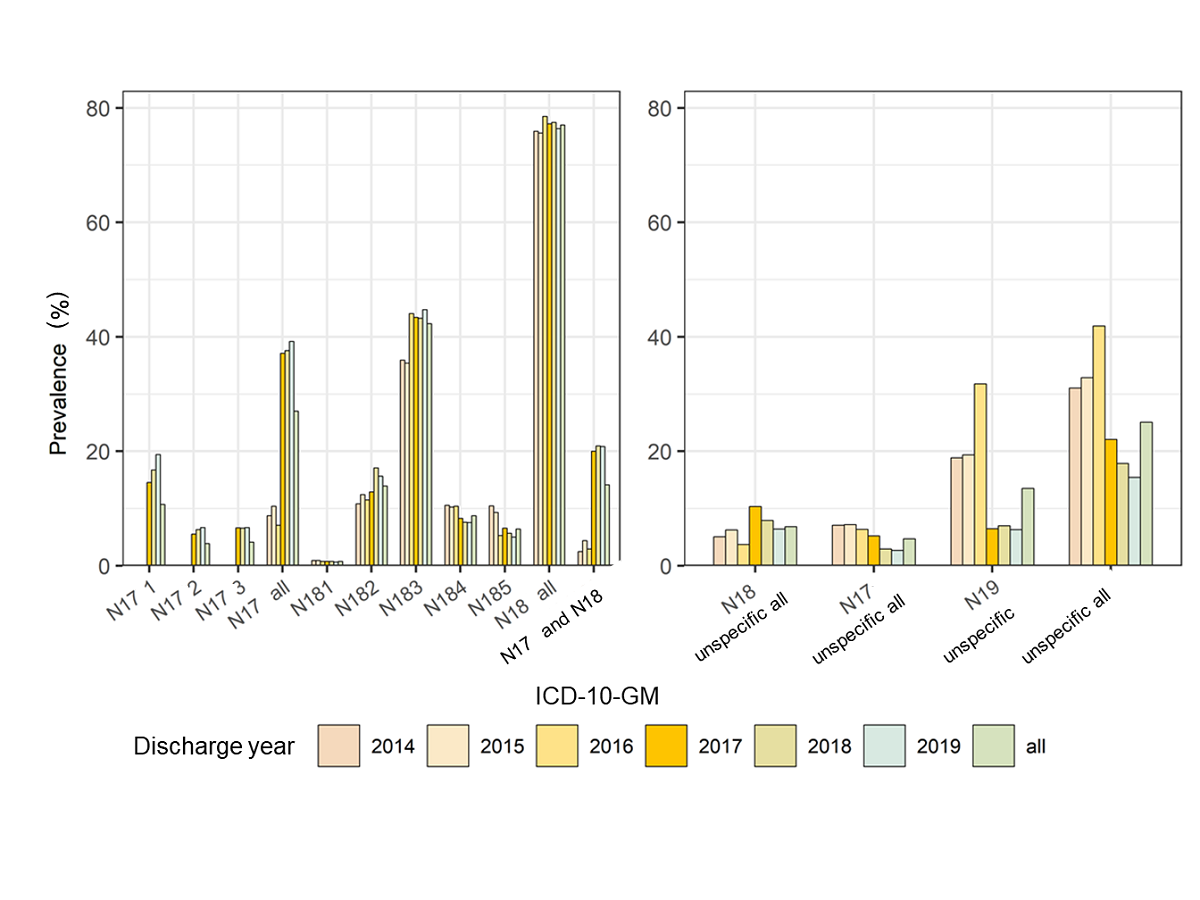
Proportion of specific ICD KD codes in all cases coded with any ICD code for KD (KD-coded cases). A: data for specific codes; B: data for unspecific codes. ICD: International Classification of Diseases; ICD-10-GM: International Statistical Classification of Diseases and Related Health Problems, 10th revision, German Modification; KD: kidney disease.

Figure 5 highlights an increase in cases with specifically coded diagnosis (ICD codes N17-/N18- with staging) and acute-on-chronic KD (ICD codes N17-/N18-) in 2014-2019 of all KD-coded cases, for example, acute-on-chronic KD cases increased from 0.26% (111/42,703) cases in 2014 and 2.62% (1706/65,146) cases in 2017 to 3.47% (2320/66,958) cases in 2019 (Multimedia Appendices 3 and 4).

Correspondingly, unspecified diagnoses (ICD N19) decreased from 2014 to 2019 (see all prevalence data in Multimedia Appendices 3 and 4 and Figure 5). A sharp decrease can be observed among 2016, 2017 (onset of the project), and 2019 in the proportion of all unspecified diagnoses (all N17-, N18-, and N19-, without staging) of all KD-coded cases, that is, 41.91% (3530/8422) cases in 2016, 22.1% (1881/8512) cases in 2017, and 15.46% (1720/11,124) cases in 2019 (Multimedia Appendices 3 and 4 and Figure 5).

Moreover, the mutation of unspecified diagnoses (ie, ICD N19-) to more precise coding (ICD N17- for AKI and N18-for CKD, including stages) during the process of diagnosis of individual cases can be demonstrated, for example, the conversion of ICD N19- to more specific codes, rising in 2017. Tables 2 and 3 illustrate the impact on the prevalence of ICD N19-.

**Table 2:** Impact of manual validation—conversion of unspecific N19 codes to specific codes and the rejection of any International Classification of Diseases coding.

Discharge year	N19 codes generated, N	N17 codes from the N19 codes generated, n (%)	N18 codes from the N19 codes generated, n (%)	N19 codes from the N19 codes generated, n (%)
2014	2967	188 (6.34)	1705 (57.47)	357 (12.03)
2015	6279	475 (7.56)	3126 (49.78)	684 (10.89)
2016	7833	460 (5.87)	3924 (50.10)	1544 (19.71)
2017	7605	2027 (26.65)	3831 (50.37)	301 (3.96)
2018	7560	2420 (32.01)	4571 (60.46)	467 (6.18)
2019	9501	3204 (33.72)	5894 (62.04)	416 (4.38)

**Table 3:** Prevalence of cases with estimated glomerular filtration rates (eGFR) of <60 ml/min and unspecified kidney injury (KI) coding (International Classification of Diseases [ICD] N19) compared with those from 2016.

Year of discharge	Cases with an eGFR of <60 ml/min, N	KI-coded (ICD N19-) cases with an eGFR of <60 ml/min, n (%)
2016	11,100	1544 (13.91)
2017^a^	10,695	301 (2.81)
2018	10,570	467 (4.42)
2019	12,519	416 (3.32)

^a^Start of the project.

**Figure 6:**
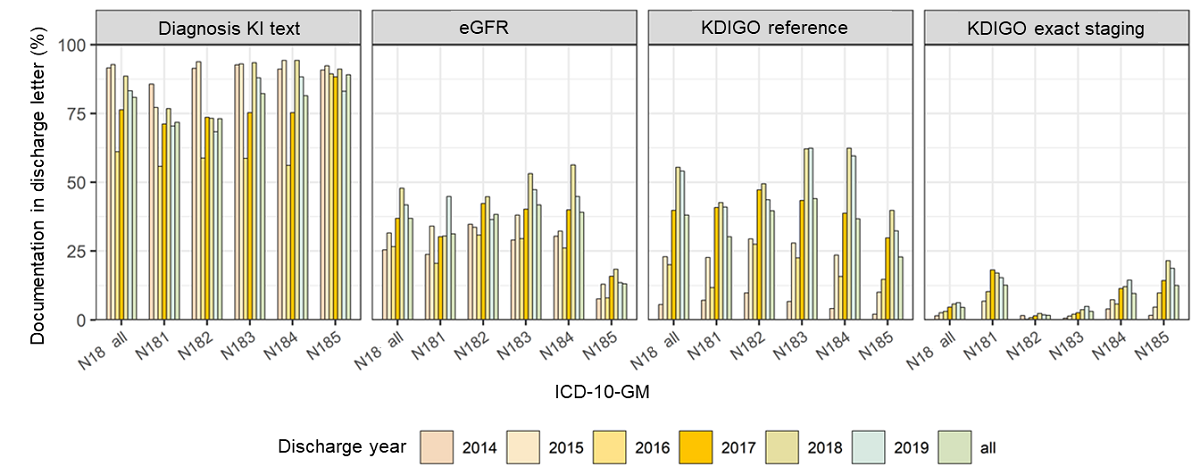
Proportion of positive text mining results in discharge letters of International Classification of Diseases N18–coded cases (chronic kidney disease). eGFR: estimated glomerular filtration rate; ICD-10-GM: International Statistical Classification of Diseases and Related Health Problems, 10th revision, German Modification; KDIGO: Kidney Disease: Improving Global Outcomes; KI: kidney injury.

Regarding discharge documentation, we observed an increase in the proportion of documented diagnoses for some KD code categories but mostly an increase in references to the KDIGO classification mentioning eGFR and SCr. Concerning the cases with coded CKD, the correct KDIGO staging could be detected more often, with all ICD N18- coded cases being 1.5% in 2014, 4.7% in 2017, and 6.3% in 2019 (Figure 6 and Multimedia Appendix 5).

**Figure 7:**
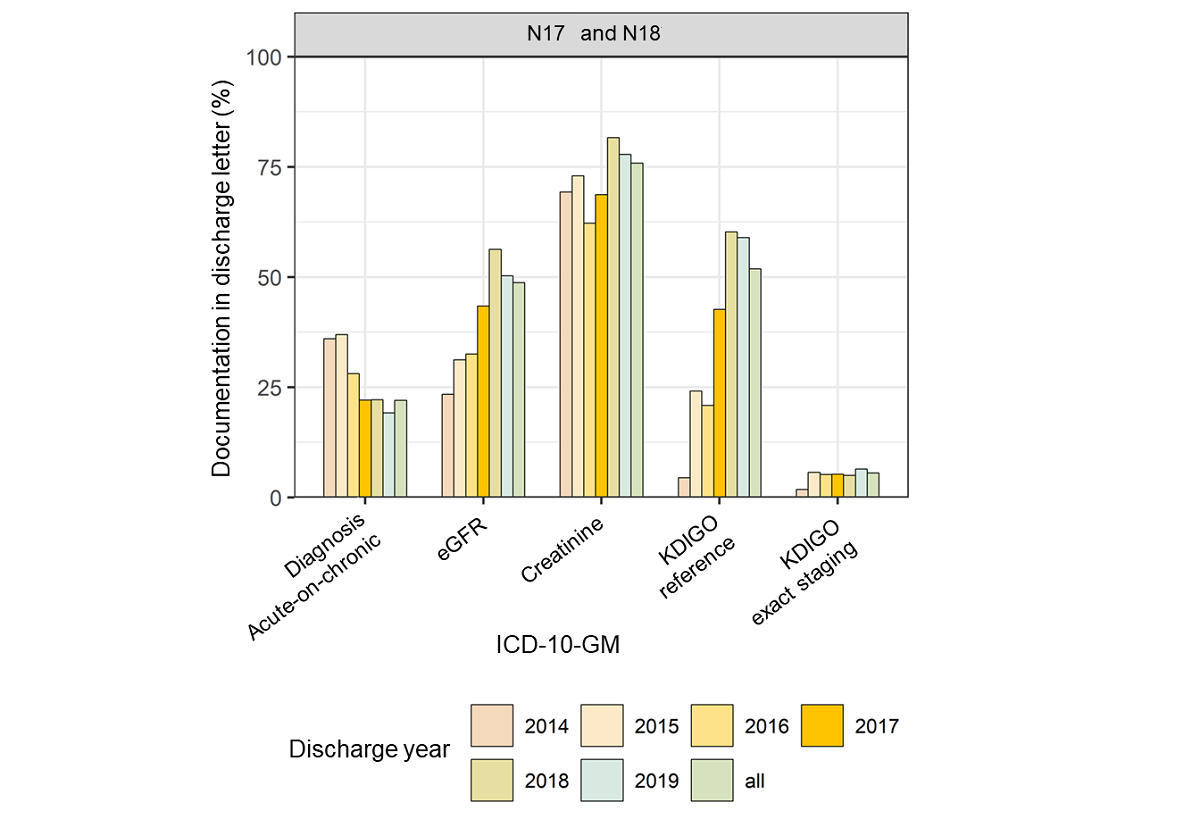
Proportion of positive text mining results in discharge letters of International Classification of Diseases N17/N18–coded cases (acute-on-chronic kidney disease). eGFR: estimated glomerular filtration rate; ICD-10-GM: International Statistical Classification of Diseases and Related Health Problems, 10th revision, German Modification; KDIGO: Kidney Disease: Improving Global Outcomes.

**Figure 8:**
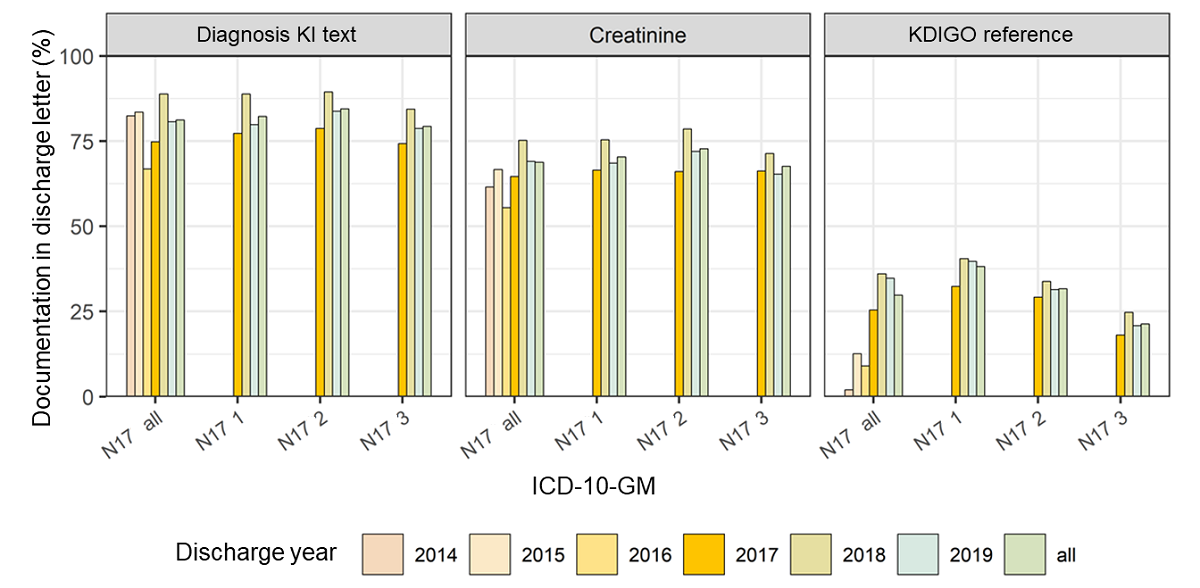
Proportion of positive text mining results in the discharge letters of International Classification of Diseases N17–coded cases (acute kidney injury). ICD-10-GM: International Statistical Classification of Diseases and Related Health Problems, 10th revision, German Modification; KI: kidney injury; KDIGO: Kidney Disease: Improving Global Outcomes.

Regarding the diagnosis of acute-on-chronic KD, a drop in documentation of the textual diagnosis could be observed at the onset of the project. Nevertheless, the SCr, eGFR, and KDIGO references were documented more often (Figure 7 and Multimedia Appendix 6). The results for AKI are shown in Figure 8 and Multimedia Appendix 7.

The effect on SwissDRG income after successful implementation of this approach accounted for a case-mix difference of 198.87 points analyzing the relevant inpatient cases (5877) from February 2020 to July 2020. Multiplied by the current standard base rate (CHF 10,800 [Swiss francs]; US $11,800), this results in CHF 2,147,753 (US $2,337,700) for this period (Table 4).

**Table 4:** Delta income of Swiss Diagnosis Related Groups, Inpatient Tariff from February 2020 to July 2020 owing to the automatization of the International Classification of Diseases (ICD) of kidney disease (KD).

Characteristics	Values
Period for inpatient cases	02/01/2020 to 07/31/2020
Cases, N	28,314
Case diagnoses^a^, n (%)	5876 (20.75)
CM^b^ with ICD, n	14,340.08
CM without ICD, n	14,141.21
Delta CM, n	198.87
Delta Swiss francs^c^, CHF	2,147,752.80^d^

^a^Any ICD diagnosis of KD automatically generated and validated afterward.

^b^CM: casemix.

^c^Standard base rate USD $11,800.

^d^USD $2,337,700.

## Discussion

### Principal Findings

After introducing the algorithm to apply AKI or CKD diagnoses, we observed an increase in the number of ICD-coded diagnoses and a shift toward higher precision in the applied stages of the diseases. Correspondingly, the number of unspecifically coded diagnoses (ICD N19-) dropped. Moreover, the documentation also improved (the correct KDIGO staging of CKD for all ICD N18- coded cases was 1.5% in 2014 and 6.3% in 2019).

### Strengths of the Project

Most studies concerning an algorithm to apply AKI or CKD diagnoses and stages consider only one diagnosis, either AKI or CKD [28,29,31]. As our project combines the 2 diagnostic criteria formulated by the KDIGO for both AKI and CKD, it improves the validity of diagnosis and enables the clinicians to easily recognize acute-on-chronic KD.

By referring to the same data set when testing for both AKI and CKD diagnoses, consistency could be improved.

This project established a link to the acknowledged impact on health at discharge by involving a defined process of validation that is conducted by text mining and communication with the clinician for retrospectively defining the exact diagnosis and staging. The process of validation of automatically generated diagnoses resulted in a decrease of unspecific diagnoses both in coding and documentation and therefore had a practical impact on the clinician’s work and on the SwissDRG income.

Many projects conducted so far have been limited to outpatients, causing a bias when calculating the overall prevalence. In particular, severe stages associated with underlying morbidities treated in inpatient care might not be recognized [10,18,24,34,42,43]. The availability of inpatient and outpatient data from the previous 6 years stored in the Insel Data Platform for all patients offered the advantage of calculating the diagnoses and the disease stages for the inpatients.

### Limitations

The validation of ICD diagnoses was strongly aimed at ICD codes with an impact on reimbursement, resulting in a bias toward validating cases with potentially higher income. Consecutively, the exact staging of diagnoses was limited to this group. Furthermore, the KDIGO classification of CKD grade 1 could not be considered for technical reasons (no limiting value of eGFR defined by KDIGO). Data on inpatient cases of all Insel Gruppe sites are available only for the years 2017-2019. Therefore, we could neither benchmark outpatient cases nor compare the data with the data of previous years. The study was limited to the description of the impact of the automatization of diagnosis; it was not designed to compare methods concerning the impact of the ACR or to determine the baseline SCr.

### Algorithm and Precision of Diagnosis

As the data required contains only laboratory results, the time stamp of the taken samples, and a patient and case identifier, the algorithms for both AKI and CKD that are presented here are transferable and ready to use.

Moreover, the process of diagnosis is facilitated and staging as a time-consuming back-calculation can be automated instead. As the algorithm applies criteria of both diagnoses separately at a specific point of time for the same case, cases with the calculated diagnosis of acute-on-chronic KD can be easily extracted to evaluate an unstable CKD versus AKI in the clinical context.

A weakness of the algorithm caused by the classifications themselves lies in the definition of CKD grade 1 according to KDIGO and ICD N18.1. As no upper value of eGFR is set, the formal testing of the data produces no sensible results. The diagnosis of CKD stage 1 can be defined only with an effective diagnosis.” [17,38].

The results displayed show the impact on (1) the increasing number of diagnoses and (2) the increasing precision in staging, documentation, and ICD coding. The higher validity and precision of diagnosis will not only improve the quality of documentation and data but also specific and timely treatment when integrated into a decision support system [30,32,34]. As the findings are translated into ICD-10 codes within the algorithm and the data of diagnosis and stage are stored, as encoded by ICD, the algorithm and the data extracted support international benchmarking and quality control by standardized diagnoses.

### Baseline

The absence of a shared approach to baseline SCr definitions [22,24,42,44] and an inter- and intraindividual and technical variability has resulted in a variability among centers regarding the interpretations for diagnosis and classification. The use of inpatient creatinine measurements as surrogates for baseline function resulted in misclassification, and the use of a minimum SCr value as a baseline inflated disease incidence [44]. In contrast to an imputed or minimum SCr value, use of the admission SCr value as a baseline resulted in nearly 50% reduction in the reported incidence of AKI compared with that of using a known outpatient baseline value [12,24,25]. This decrease is perhaps best explained by the missed diagnosis of community-acquired AKI that improves during hospitalization. The higher mortality rates observed when using this baseline reflect the bias of using this method, which is only sensitive to AKI that continues to worsen during hospitalization. Because of this lack of joint approach to baseline SCr definitions and lack of other markers, we specified the following to reflect the patients’ premorbid state: either baseline SCr is the lowest value during hospitalization or the arithmetic mean of all outpatient SCr measurements 90 days before the index admission (relying on the lowest value of both methods for diagnosis) is the lowest value.

The comparability of studies concerning AKI, including this project, might be impaired regarding the baseline definition, a weakness that can only to be resolved by additional consensus criteria to better characterize preadmission AKI and by specifying a standard method to incorporate previously known baseline data. Being aware of the potential inflation of diagnosis when using the lowest inpatient SCr [44], the approach would ensure a higher sensitivity regarding the clinician’s awareness.

### Electronic Health Records

Integrating data and computer-based entries into electronic health records may support precision, standardization, and decision regarding patients’ health care and lead to a more specific, valid, reliable, and consistent database. Greater data integration may also provide information not only for timely treatment but also disease registries and clinical trials [15,28,30,31,33,34]. Automated decision support based on arithmetic algorithms may be too rigid. Therefore, with the experience gained from this project, we favor an integrated solution that closes the loop between an automated alert and clinicians’ validation. As demonstrated in this study, part of the validation can be automated by text mining to minimize the workload [30]. However, many cases require manual validation. Lack of documentation as seen in the inpatient cases of 2016 (fusion of data and documentation of all Insel Gruppe sites) compromises automatization.

### Lessons Learned and Future Work

The project will be an important achievement for inpatient and outpatient care, especially with chronic diseases, such as CKD and acute-on-chronic KD and its complex algorithm. As the prevalence of KD is underestimated [7,8,11], the higher validity and precision of diagnosis will not only improve the reliability of documentation and data but will also improve treatment and reimbursement. This will result in the efficiency and quality of the diagnosis process, a higher reliability, and a highly standardized database.

The difficulty of defining the right baseline for AKI could not finally be solved. Missing values before admission should be addressed and anticipated. Clinicians and other medical experts should be closely involved in the process of setting up requirements and validating diagnoses.

This project introduced an end-to-end approach to clinical decision support at the Insel Gruppe hospitals.

## Appendix

Multimedia Appendix 1 Algorithm for all diagnoses.

Multimedia Appendix 2 International Statistical Classification of Diseases and Related Health Problems, 10th revision, German Modification (ICD-10-GM) code catalogs from 2012 to 2018 that were effective in Switzerland from 2014 to 2020.

Multimedia Appendix 3 Counts of cases with N17 coded.

Multimedia Appendix 4 Counts of cases with N18, N19, acute-on-chronic, unspecified kidney injury, and kidney disease (KI/KD) coded.

Multimedia Appendix 5 Proportion of International Classification of Diseases (ICD)–coded cases N18- with documentation in the discharge letter.

Multimedia Appendix 6 Proportion of International Classification of Diseases (ICD)–coded cases N17- and 18- acute-on-chronic with documentation in discharge letter.

Multimedia Appendix 7 Proportion of International Classification of Diseases (ICD)–coded cases N17- with documentation in the discharge letter.

## Abbreviations

ACR: albumin creatinine ratio
AKI: acute kidney injury
CKD: chronic kidney disease
CKD-EPI: Chronic Kidney Disease Epidemiology Collaboration
eGFR: estimated glomerular filtration rate
GFR: glomerular filtration rate
ICD: International Classification of Diseases
ICD-10-GM: International Statistical Classification of Diseases and Related Health Problems, 10th revision, German Modification
KD: kidney disease
KDIGO: Kidney Disease: Improving Global Outcomes
NHS: National Health Service
SCr: serum creatinine
SwissDRG: Swiss Diagnosis Related Groups, Inpatient Tariff
