# Supplementary material for: Real-world Health Data and Precision for the Diagnosis of Acute Kidney Injury, Acute-on-Chronic Kidney Disease, and Chronic Kidney Disease: Observational Study
Source: JMIR Med Inform. 2022 Jan 25;10(1):e31356. doi: 10.2196/31356 (PMC8826149; doi:10.2196/31356)
Supplement: Multimedia Appendix 1 [file medinform_v10i1e31356_app1.pptx]

## Slide 1
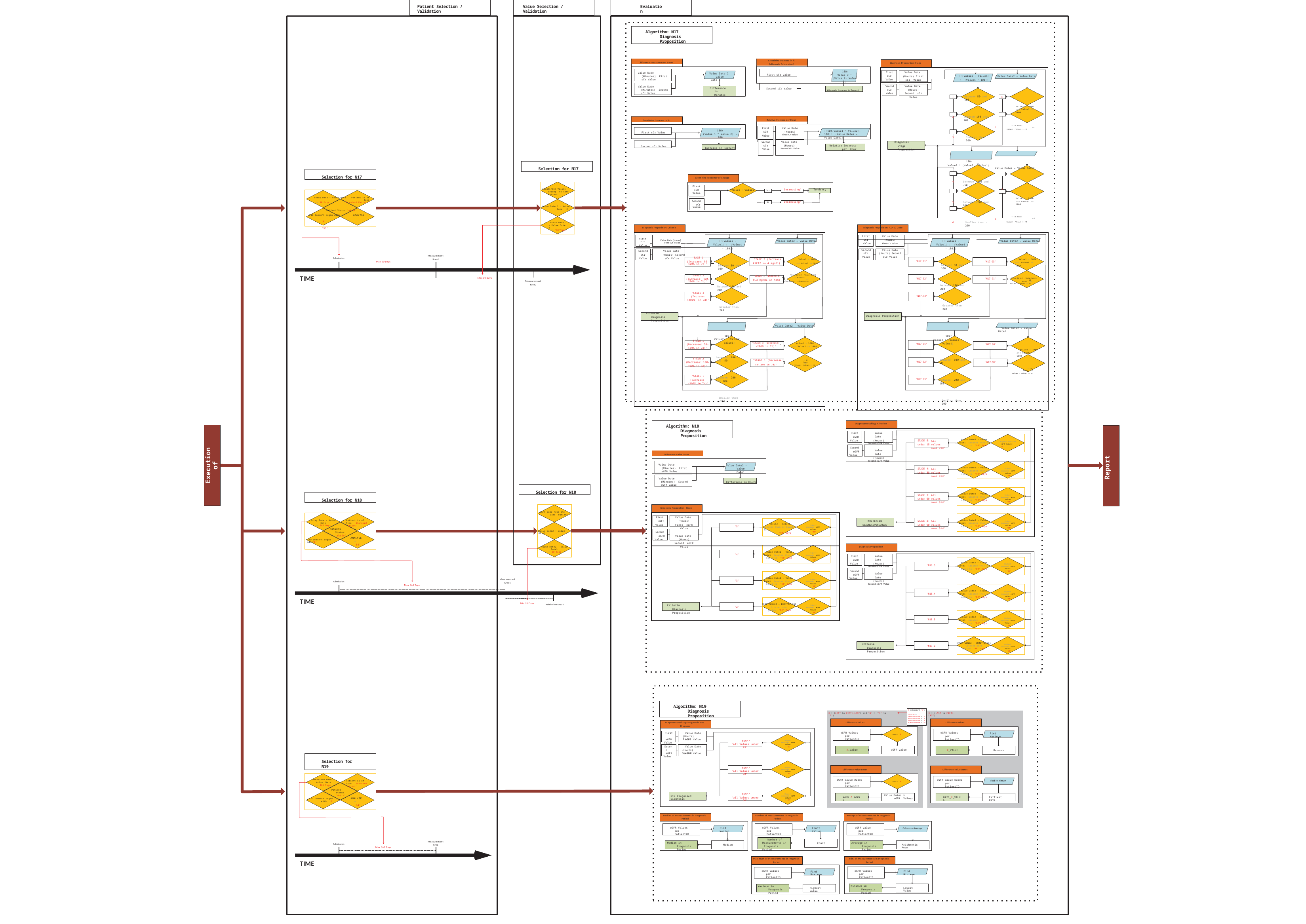

Patient Selection / Validation
Value Selection / Validation
Evaluation
Algorithm: N17 Diagnosis Proposition
Difference Measurement Dates
Creatinine Increase in % (alternate Calculation)
| Diagnosis Proposition: Stage | | | |
| --- | --- | --- | --- |
| First sCr Value | Value Date (Hours) First sCr Value | (((Value2 – Value1) / (Value1 \* 100)) Between 50 and 100 between 100 and 200 Greater than 200 100/ Value2 \* (Value2 – Value1) between -100 and -50 between -200 and 100 Smaller than -200 | Value Date2 – Value Date1 3 Value1 < 1000 und Value2 >= 1000 <= 48 Hours 1 and Value2 – Value1 >= 75 Value Date2 – Value Date1 3 Value1 > 1000 and Value2 <= 1000 <= 48 Hours 1 and Value2 – Value1 >= 75 |
| Second sCr Value | Value Date (Hours) Second sCr Value | | |
| | Diagnosis Stage Proposition | | |
100/
Value 2 *
(Value 2– Value
1)
Value Date (Minutes) First sCr Value
First sCr Value
Value Date 2 – Value Date 1
Value Date (Minutes) Second sCr Value
Second sCr Value
Difference in Minutes
Alternate Increase in Percent
Relative Increase per Hour
Creatinine Increase in %
((100/Value1 * Value2)-100)/ (Value Date2 – Value Date1)
100/
(Value 1 * Value 2) -
100
| First sCR Value | | Value Date (Hours) First sCr Value |
| --- | --- | --- |
| | | |
First sCr Value
| Second sCr Value | | Value Date (Hours) Second sCr Value |
| --- | --- | --- |
| | | |
Second sCr Value
Relative Increase per Hour
Increase in Percent
Selection for N17
Selection for N17
| Creatinine Tendency of Change | | | | | | | |
| --- | --- | --- | --- | --- | --- | --- | --- |
| First | | | | | | | |
| sCR | Value1 | | < | Value2 | Yes | 'Increasing' | Tendency |
| Value | | | | | | | |
| Second sCr | | | | | No | 'Decreasing' | |
| Value | | | | | | | |
Creatinine Values Belong to Same Patient
Entry Date – Value Date	Patient is of Type
<= 30 Days	'Standard Patient'
Patient Status 'aktiv'
Value Date 1 < Value Date 2
FID Doesn’t begin with	ANALYSE =
'E'	'53'
Value Date 2
- Value Date 1
<= '30'Tage
| Diagnosis Proposition: Criteria | | | |
| --- | --- | --- | --- |
| First Value Date (Hours) sCr First sCr Value Value | | (((Value2 – Value1) / (Value1 \* 100)) Between 50 and 100 Between 100 and 200 Greater than 200 100/ Value2 \* (Value2 – Value1) between -100 and -50 between -200 and 100 Smaller than -200 | Value Date2 – Value Date1 'STAGE 3 (Increase: Value1 < 1000 KREA2 >= 4 mg/dl)' und Value2 >= 1000 'STAGE 1 (Increase: Value Date2 – Value Date1 <= 48 Hours 0.3 mg/dl in 48h)' and Value Date2 – Value Date1 >= 75 Value Date2 – Value Date1 'STAGE 3 (Decrease: Value1 > 1000 >200% in 7d)' and Value2 <= 1000 'STAGE 1 (Decrease: <= 48 Hours and 50-100% in 7d)' Value2 - Value1 >= 75 |
| Second Value Date sCr (Hours) Second Value sCr Value | | | |
| Criteria Diagnosis Proposition | | | |
| | 'STAGE 1 (Decrease: 50- 100% in 7d)' | | |
| | 'STAGE 2 (Decrease: 100- 200% in 7d)' | | |
| | 'STAGE 3 (Decrease: >200% in 7d)' | | |
| Diagnosis Proposition: ICD-10 Code | | | | |
| --- | --- | --- | --- | --- |
| First Value Date | | (((Value2 – Value1) / (Value1 \* 100)) between 50 and 100 between 100 and 200 Greater than 200 100/ Value2 \* (Value2 – Value1) between -100 and -50 between -200 and 100 Smaller than -200 | | Value Date2 – Value Date1 Value1 < 1000 und Value2 >= 1000 Value Date2 – Value DAte1 <= 48 Hours und Value2 – Value1 >= 75 Value Date2 – Value Date1 Value1 > 1000 and Value2 <= 1000 <= 48 Hours and Value2 - Value1 >= 75 |
| sCr (Hours) | | | | |
| Value First sCr Value | | | | |
| Second Value Date | | | | |
| sCr (Hours) Second | | | | |
| Value sCr Value | | | | |
| | 'N17.91' | | 'N17.93' | |
| | 'N17.92' | | 'N17.91' | |
| | 'N17.93' | | | |
| Diagnosis Proposition | | | | |
| | 'N17.91' | | 'N17.93' | |
| | 'N17.92' | | 'N17.91' | |
| | 'N17.93' | | | |
Measurement Krea1
Admission
| 'SAGE 1 (Increase: 50- 100% in 7d)' | |
| --- | --- |
| | |
| 'STAGE 2 (Increase: 100- 200% in 7d)' | |
| | |
| 'STAGE 3 (Increse: >200% in 7d)' | |
| | |
Max 30 Days
TIME
Max 30 Days
Measurement Krea2
Algorithm: N18 Diagnosis Proposition
| Diagnosevorschlag: Kriterien | | | | | | |
| --- | --- | --- | --- | --- | --- | --- |
| First eGFR Value Second eGFR Value | Value Date (Hours) Second eGFR Value Value Date (Hours) Second eGFR Value | 'STAGE under | 5: 15 | All values over 91d' | Value Date2 – Value Date1 Greater than or equal to '90' Days | Highest measured eGFR Value1< '15' |
| | | 'STAGE under | 4: 30 | All values over 91d' | Value Date2 – Value Date1 Greater than or equal to '90' Days | Highest measured eGFR Value1 < '30' |
| | | 'STAGE under | 3: 60 | All values over 91d' | Value Date2 – Value Date1 Greater than or equal to '90' Days | Highest measured eGFR Value1 < '60' |
| | KRITERIEN\_ DIAGNOSEVORSCHLAG | 'STAGE under | 2: 90 | All values over 91d' | Value Date2 – Value Date1 Greater than or equal to '90' Days | Highest measured eGFR Value1 < '60' |
Report
Execution of
Algorithm
Difference Value Dates
Value Date2 – Value Date1
Value Date (Minutes) First eGFR Value
Value Date (Minutes) Second eGFR Value
Difference in Hours
Selection for N18
Selection for N18
| Diagnosis Proposition: Stage | | | | |
| --- | --- | --- | --- | --- |
| First eGFR Value Second eGFR Value | Value Date (Hours) First eGFR Value Value Date (Hours) Second eGFR Value | '5' | Value2 – Value1 Greater than or Equal to '90' Days | Highest measured eGFR Value1 < '15' |
| | | '4' | Value Date2 – Value Date1 Greater than or Equal to '90' Days | Highest measured eGFR Value1 < '30' |
| | | '3' | Value Date2 – Value Date1 Greater than or Equal to '90' Days | Highest measured eGFR Value1 < '60' |
| | Criteria Diagnosis Proposition | '2' | ERMITTLUNG2 – ERMITTLUNG1 Grösser oder gleich '90' Tage | Highest measured eGFR Value1 < '60' |
eGFR Come From the Same Patient
Patient is of Type 'Standard Patient'
Entry Date – Value Date
<= 365 Days
Patient Status 'aktiv'
Value Date2 > Value Date1
ANALYSE = '64'
FID Doesn’t begin with'E'
| Diagnosis Proposition | | | | |
| --- | --- | --- | --- | --- |
| First eGFR Value Second eGFR Value | Value Date (Hours) Second eGFR Value Value Date (Hours) Second eGFR Value | 'N18.5' | Value Date2 – Value Date1 Greater than or equal to '90' Days | Highest measured eGFR Value1 < '15' |
| | | 'N18.4' | Value Date2 – Value Date1 Greater than or equal to '90' Days | Highest measured eGFR Value1 < '30' |
| | | 'N18.3' | Value Date2 – Value Date1 Greater than or equal to '90' Days | Highest measured eGFR Value1 < '60' |
| | Criteria Diagnosis Proposition | 'N18.2' | ERMITTLUNG2 – ERMITTLUNG1 Grösser oder gleich '90' Days | Highest measured eGFR Value1 < '60' |
Value Date2 – Value Date1
>= 90 Tage
Measurement Krea1
Admission
Max 365 Tage
TIME
Min 90 Days
Admission Krea2
Algorithm: N19 Diagnosis Proposition
X entspricht 'N';
X = (LAST to FIFTH-LAST) and 'N' = ('1' to '5')
X = (LAST to FIFTH-LAST):
LETZTER = '1'
ZWEITLETZTER = '2'
DRITTLETZTER = '3'
VIERTLETZTER = '4'
Difference Values
Diagnosevorschlag: Prognostizierte Diagnose
Difference Values
FUNFTLETZTER = '5'
eGFR Values per PatientID
eGFR Values per PatientID
Value Date (Hours) First
First eGFR
Value
Find Maximum
Row = 'N'
eGFR Value
'N19'/
'all Values under 15'
Highest measured eGFR Value1
< '15'
Value Date (Hours) Second
Second eGFR
Value
X_Value
eGFR Value
X_VALUE
Maximum
eGFR Value
Selection for N19
'N19'/
'all Values under 30'
Highest measured eGFR Value1
< '30'
Difference Value Dates
Difference Value Dates
Admission Date – Value Date
<= 365 Tage
eGFR Value Dates per PatientID
eGFR Value Dates per PatientID
Find Minimum
Patient is of Type 'Standard Patient'
Row = 'N'
Patient status 'aktiv'
'N19'/
'all Values under 60'
Highest measured eGFR Value1
< '60'
Value Dates v. eGFR Values
N19 Prognosed Diagnosis
DATE_X_VALUE
DATE_X_VALUE
Earliest Date
ANALYSE = '64'
FID Doesn’t begin with 'E'
Median of Measurements in Prognosis Period
Number of Measurements in Prognosis Perion
Average of Measurements in Prognosis Period
eGFR Values per PatientID
eGFR Values per PatientID
eGFR Value per PatientID
Find Median
Count Values
Calculate Average
Number of Measurements in Prognosis Period
Measurement Krea
Median in Prognosis Period
Average in Prognosis Period
Count
Admission
Median
Arithmetic Mean
Max 365 Days
Min. of Measurements in Prognosis Period
Maximum of Measurements in Prognosis Period
TIME
eGFR Values per PatientID
eGFR Values per PatientID
Find Minimum
Find Maximum
Minimum in Prognosis Period
Maximum in Prognosis Period
Lowest Value
Highest Value
