# Supplementary material for: Real-world Health Data and Precision for the Diagnosis of Acute Kidney Injury, Acute-on-Chronic Kidney Disease, and Chronic Kidney Disease: Observational Study
Source: JMIR Med Inform. 2022 Jan 25;10(1):e31356. doi: 10.2196/31356 (PMC8826149; doi:10.2196/31356)
Supplement: Multimedia Appendix 2 [file medinform_v10i1e31356_app2.docx]

Multimedia Appendix 2: ICD-10_GM codes catalogues from 2012 - 2018, effective in CH 2014 – 2020

| Code | Text (German) | Discharge year | KDIGO staging |
| --- | --- | --- | --- |
|  |  |  |  |
| N17.0 | Akutes Nierenversagen mit Tubulusnekrose | 2014 - 2016 | non |
| N17.1 | Akutes Nierenversagen mit akuter Rindennekrose | 2014 - 2016 | non |
| N17.2 | Akutes Nierenversagen mit Marknekrose | 2014 - 2016 | non |
| N17.01 | Akutes Nierenversagen mit Tubulusnekrose, Stadium 1 | 2017 - 2019 | AKI 1 |
| N17.02 | Akutes Nierenversagen mit Tubulusnekrose, Stadium 2 | 2017 - 2019 | AKI 2 |
| N17.03 | Akutes Nierenversagen mit Tubulusnekrose, Stadium 3 | 2017 - 2019 | AKI 3 |
| N17.09 | Akutes Nierenversagen mit Tubulusnekrose | 2017 - 2019 | non |
| N17.11 | Akutes Nierenversagen mit akuter Rindennekrose, Stadium 1 | 2017 - 2019 | AKI 1 |
| N17.12 | Akutes Nierenversagen mit akuter Rindennekrose, Stadium 2 | 2017 - 2019 | AKI 2 |
| N17.13 | Akutes Nierenversagen mit akuter Rindennekrose, Stadium 3 | 2017 - 2019 | AKI 3 |
| N17.19 | Akutes Nierenversagen mit akuter Rindennekrose | 2017 - 2019 | non |
| N17.21 | Akutes Nierenversagen mit Marknekrose, Stadium 1 | 2017 - 2019 | AKI 1 |
| N17.22 | Akutes Nierenversagen mit Marknekrose, Stadium 2 | 2017 - 2019 | AKI 2 |
| N17.23 | Akutes Nierenversagen mit Marknekrose, Stadium 3 | 2017 - 2019 | AKI 3 |
| N17.29 | Akutes Nierenversagen mit Marknekrose | 2017 - 2019 | non |
| N17.81 | Sonstiges akutes Nierenversagen, Stadium 1 | 2017 - 2019 | AKI 1 |
| N17.82 | Sonstiges akutes Nierenversagen, Stadium 2 | 2017 - 2019 | AKI 2 |
| N17.83 | Sonstiges akutes Nierenversagen, Stadium 3 | 2017 - 2019 | AKI 3 |
| N17.89 | Sonstiges akutes Nierenversagen | 2017 - 2019 | non |
| N17.91 | Akutes Nierenversagen, nicht näher, Stadium 1 | 2017 - 2019 | AKI 1 |
| N17.92 | Akutes Nierenversagen, nicht näher, Stadium 2 | 2017 - 2019 | AKI 2 |
| N17.93 | Akutes Nierenversagen, nicht näher, Stadium 3 | 2017 - 2019 | AKI 3 |
| N17.99 | Akutes Nierenversagen, nicht näher | 2017 - 2019 | non |
| N17.8 | Sonstiges akutes Nierenversagen | 2014 - 2016 | non |
| N17.9 | Akutes Nierenversagen, nicht näher bezeichnet | 2014 - 2016 | non |
| N18.1 | Chronische Nierenkrankheit, Stadium 1 | 2014 - 2019 | CKD 1 |
| N18.2 | Chronische Nierenkrankheit, Stadium 2 | 2014 - 2019 | CKD 2 |
| N18.3 | Chronische Nierenkrankheit, Stadium 3 | 2014 - 2019 | CKD 3 |
| N18.4 | Chronische Nierenkrankheit, Stadium 4 | 2014 - 2019 | CKD 4 |
| N18.5 | Chronische Nierenkrankheit, Stadium 5 | 2014 - 2019 | CKD 5 |
| N18.80 | Einseitige chronische Nierenfunktionsstörung | 2014 - 2019 | non |
| N18.89 | Sonstige chronische Nierenkrankheit, Stadium nicht näher bezeichnet | 2014 - 2019 | non |
| N18.9 | Chronische Nierenkrankheit, nicht näher bezeichnet | 2014 - 2019 | non |
| N19 | Sonstige chronische Nierenkrankheit, Stadium nicht näher bezeichnet | 2014 - 2019 | non |
