# Supplementary material for: Real-world Health Data and Precision for the Diagnosis of Acute Kidney Injury, Acute-on-Chronic Kidney Disease, and Chronic Kidney Disease: Observational Study
Source: JMIR Med Inform. 2022 Jan 25;10(1):e31356. doi: 10.2196/31356 (PMC8826149; doi:10.2196/31356)
Supplement: Multimedia Appendix 3 [file medinform_v10i1e31356_app3.docx]

Multimedia Appendix 3: Count N17

|  | discharge year | | | | | | |
| --- | --- | --- | --- | --- | --- | --- | --- |
| count cases | 2014 | 2015 | 2016 | 2017 | 2018 | 2019 | all |
| all | 42703 | 45138 | 64478 | 65146 | 66038 | 66958 | 350461 |
| KI coded all | 4491 | 4786 | 8422 | 8512 | 10165 | 11124 | 47500 |
| N17.0 | 25 | 29 | 44 |  |  |  | 98 |
| N17.01 |  |  |  | 4 | 11 | 2 | 17 |
| N17.02 |  |  |  | 5 | 12 | 8 | 25 |
| N17.03 |  |  |  | 28 | 26 | 25 | 79 |
| N17.09 |  |  |  | 6 | 2 | 1 | 9 |
| N17.1 | 3 | 3 |  |  |  |  | 6 |
| N17.11 |  |  |  | 2 | 5 |  | 7 |
| N17.12 |  |  |  | 1 |  | 1 | 2 |
| N17.13 |  |  |  | 3 |  |  | 3 |
| N17.8 | 136 | 166 | 241 |  |  |  | 543 |
| N17.81 |  |  |  | 72 | 8 | 8 | 88 |
| N17.82 |  |  |  | 33 | 4 | 8 | 45 |
| N17.83 |  |  |  | 75 | 5 | 12 | 92 |
| N17.89 |  |  |  | 26 | 2 | 2 | 30 |
| N17.9 | 229 | 301 | 313 |  |  |  | 843 |
| N17.91 |  |  |  | 1162 | 1680 | 2152 | 4994 |
| N17.92 |  |  |  | 433 | 628 | 726 | 1787 |
| N17.93 |  |  |  | 458 | 638 | 707 | 1803 |
| N17.99 |  |  |  | 852 | 801 | 712 | 2365 |
| N17 unspec | 229 | 301 | 313 | 884 | 805 | 715 | 3247 |
| N17*1 all | 0 | 0 | 0 | 1240 | 1704 | 2162 | 5106 |
| N17*2 all | 0 | 0 | 0 | 472 | 644 | 743 | 1859 |
| N17*3 all | 0 | 0 | 0 | 564 | 669 | 744 | 1977 |
