# Supplementary material for: Real-world Health Data and Precision for the Diagnosis of Acute Kidney Injury, Acute-on-Chronic Kidney Disease, and Chronic Kidney Disease: Observational Study
Source: JMIR Med Inform. 2022 Jan 25;10(1):e31356. doi: 10.2196/31356 (PMC8826149; doi:10.2196/31356)
Supplement: Multimedia Appendix 4 [file medinform_v10i1e31356_app4.docx]

Multimedia Appendix 4: Count N18, N19, acute on chronic, unspec KI/KD all

|  | discharge year | | | | | | |
| --- | --- | --- | --- | --- | --- | --- | --- |
| count | 2014 | 2015 | 2016 | 2017 | 2018 | 2019 | all |
| cases all | 42703 | 45138 | 64478 | 65146 | 66038 | 66958 | 350461 |
| cases KI coded all | 4491 | 4786 | 8422 | 8512 | 10165 | 11124 | 47500 |
| N18.1 | 42 | 44 | 68 | 66 | 82 | 78 | 380 |
| N18.2 | 488 | 597 | 970 | 1101 | 1742 | 1741 | 6639 |
| N18.3 | 1614 | 1695 | 3715 | 3694 | 4398 | 4978 | 20094 |
| N18.4 | 476 | 492 | 879 | 709 | 774 | 842 | 4172 |
| N18.5 | 471 | 446 | 447 | 559 | 580 | 558 | 3061 |
| N18.80 | 8 | 6 | 9 | 1 | 4 | 1 | 29 |
| N18.89 | 20 | 55 | 199 | 135 | 86 | 81 | 576 |
| N18.9 | 291 | 284 | 329 | 310 | 211 | 220 | 1645 |
| N18* unspec all | 319 | 345 | 537 | 446 | 301 | 302 | 2250 |
| N18* all | 3410 | 3619 | 6616 | 6575 | 7877 | 8499 | 36596 |
| N19 | 848 | 929 | 2680 | 551 | 713 | 703 | 6424 |
| cases N17*/N18* | 111 | 211 | 249 | 1706 | 2130 | 2320 | 6727 |
| cases unspec KI all | 1396 | 1575 | 3530 | 1881 | 1819 | 1720 | 11921 |
