# Supplementary material for: Real-world Health Data and Precision for the Diagnosis of Acute Kidney Injury, Acute-on-Chronic Kidney Disease, and Chronic Kidney Disease: Observational Study
Source: JMIR Med Inform. 2022 Jan 25;10(1):e31356. doi: 10.2196/31356 (PMC8826149; doi:10.2196/31356)
Supplement: Multimedia Appendix 5 [file medinform_v10i1e31356_app5.docx]

Multimedia Appendix 5: Proportion of ICD coded cases N18* with documentation in discharge letter

|  |  |  | year of discharge | | | | | | |
| --- | --- | --- | --- | --- | --- | --- | --- | --- | --- |
| documentation | | ICD code | 2014 | 2015 | 2016 | 2017 | 2018 | 2019 | all |
|  | diagnosis KI text regardless of staging | N18* all | 91.6 | 92.8 | 61.2 | 76.4 | 88.6 | 83.3 | 80.9 |
|  |  | N181 | 85.7 | 77.3 | 55.9 | 71.2 | 76.8 | 70.5 | 71.8 |
|  |  | N182 | 91.4 | 93.8 | 58.9 | 73.7 | 73.3 | 68.4 | 73.1 |
|  |  | N183 | 92.8 | 93.1 | 58.8 | 75.3 | 93.5 | 88 | 82.3 |
|  |  | N184 | 91.2 | 94.3 | 56.2 | 75.3 | 94.3 | 88.4 | 81.5 |
|  |  | N185 | 90.9 | 92.4 | 89.5 | 88.4 | 91.2 | 83.2 | 89.1 |
|  | eGFR | N18* all | 25.5 | 31.6 | 26.6 | 36.9 | 47.9 | 41.8 | 37 |
|  |  | N181 | 23.8 | 34.1 | 20.6 | 30.3 | 30.5 | 44.9 | 31.3 |
|  |  | N182 | 34.8 | 33.7 | 30.8 | 42.3 | 44.8 | 36.5 | 38.4 |
|  |  | N183 | 29.1 | 38.1 | 29.6 | 40.3 | 53.2 | 47.4 | 41.8 |
|  |  | N184 | 30.5 | 32.3 | 26.2 | 40.1 | 56.3 | 44.9 | 39.1 |
|  |  | N185 | 7.6 | 13 | 8.1 | 15.9 | 18.4 | 13.6 | 13.1 |
|  | KDIGO reference | N18* all | 5.7 | 23 | 20.1 | 39.8 | 55.5 | 54.2 | 38.1 |
|  |  | N181 | 7.1 | 22.7 | 11.8 | 40.9 | 42.7 | 41 | 30.3 |
|  |  | N182 | 9.8 | 29.5 | 27.5 | 47.3 | 49.5 | 43.7 | 39.7 |
|  |  | N183 | 6.7 | 28 | 22.6 | 43.4 | 62.2 | 62.4 | 44.1 |
|  |  | N184 | 4.2 | 23.6 | 15.8 | 38.8 | 62.4 | 59.6 | 36.8 |
|  |  | N185 | 2.1 | 10.1 | 14.8 | 29.9 | 39.8 | 32.4 | 22.9 |
|  | exact KDIGO staging | N18* all | 1.5 | 2.6 | 3.1 | 4.7 | 5.8 | 6.3 | 4.6 |
|  |  | N181 | NV | 6.8 | 10.3 | 18.2 | 17.1 | 15.4 | 12.6 |
|  |  | N182 | 1.6 | 0.3 | 0.8 | 1.5 | 2.4 | 1.9 | 1.7 |
|  |  | N183 | 0.7 | 1.4 | 2.2 | 2.7 | 3.8 | 4.9 | 3.1 |
|  |  | N184 | 4 | 7.3 | 5.8 | 11.4 | 12.1 | 14.5 | 9.7 |
|  |  | N185 | 1.7 | 4.7 | 9.8 | 14.3 | 21.6 | 18.8 | 12.5 |
