# Supplementary material for: Real-world Health Data and Precision for the Diagnosis of Acute Kidney Injury, Acute-on-Chronic Kidney Disease, and Chronic Kidney Disease: Observational Study
Source: JMIR Med Inform. 2022 Jan 25;10(1):e31356. doi: 10.2196/31356 (PMC8826149; doi:10.2196/31356)
Supplement: Multimedia Appendix 6 [file medinform_v10i1e31356_app6.docx]

Multimedia Appendix 6: Proportion of ICD coded cases N17*/18* acute-on-chronic with documentation in discharge letter

|  | year of discharge | | | | | | |
| --- | --- | --- | --- | --- | --- | --- | --- |
| documentation | 2014 | 2015 | 2016 | 2017 | 2018 | 2019 | all |
| diagnosis acute on chronic regardless of staging | 36.0 | 37.0 | 28.1 | 22.2 | 22.2 | 19.2 | 22.1 |
| eGFR | 23.4 | 31.3 | 32.5 | 43.4 | 56.3 | 50.3 | 48.8 |
| Creatinine | 69.4 | 73.0 | 62.2 | 68.7 | 81.7 | 77.8 | 75.9 |
| KDIGO reference | 4.5 | 24.2 | 20.9 | 42.7 | 60.3 | 59.0 | 51.9 |
| exact KDIGO staging | 1.8 | 5.7 | 5.2 | 5.3 | 5.0 | 6.4 | 5.5 |
