# Supplementary material for: Real-world Health Data and Precision for the Diagnosis of Acute Kidney Injury, Acute-on-Chronic Kidney Disease, and Chronic Kidney Disease: Observational Study
Source: JMIR Med Inform. 2022 Jan 25;10(1):e31356. doi: 10.2196/31356 (PMC8826149; doi:10.2196/31356)
Supplement: Multimedia Appendix 7 [file medinform_v10i1e31356_app7.docx]

Multimedia Appendix 7: Proportion of ICD coded cases N17* with documentation in discharge letter

|  |  |  | year of discharge | | | | | | |
| --- | --- | --- | --- | --- | --- | --- | --- | --- | --- |
| documentation | | ICD code | 2014 | 2015 | 2016 | 2017 | 2018 | 2019 | all |
|  | diagnosis KI text regardless of staging | N17* all | 82.4 | 83.6 | 66.9 | 74.9 | 88.8 | 80.8 | 81.2 |
|  |  | N17*1 |  |  |  | 77.3 | 88.9 | 79.9 | 82.3 |
|  |  | N17*2 |  |  |  | 78.8 | 89.4 | 83.8 | 84.5 |
|  |  | N17*3 |  |  |  | 74.3 | 84.5 | 78.8 | 79.4 |
|  | creatinine | N17* all | 61.6 | 66.7 | 55.5 | 64.6 | 75.2 | 69.1 | 68.9 |
|  |  | N17*1 |  |  |  | 66.5 | 75.4 | 68.6 | 70.4 |
|  |  | N17*2 |  |  |  | 66.1 | 78.6 | 72.0 | 72.8 |
|  |  | N17*3 |  |  |  | 66.3 | 71.5 | 65.3 | 67.7 |
|  | KDIGO reference | N17* all | 2.0 | 12.6 | 9.0 | 25.5 | 36.1 | 34.8 | 29.8 |
|  |  | N17*1 |  |  |  | 32.4 | 40.6 | 39.7 | 38.2 |
|  |  | N17*2 |  |  |  | 29.2 | 33.9 | 31.5 | 31.7 |
|  |  | N17*3 |  |  |  | 18.1 | 24.8 | 20.8 | 21.4 |
